# Pathogen Threat and In-group Cooperation

**DOI:** 10.3389/fpsyg.2021.678188

**Published:** 2021-06-29

**Authors:** Hirotaka Imada, Nobuhiro Mifune

**Affiliations:** ^1^School of Psychology, University of Kent, Canterbury, United Kingdom; ^2^School of Economics and Management, Kochi University of Technology, Kochi, Japan

**Keywords:** behavioral immune system, pathogen threat, cooperation, in-group bias, assortative sociality

## Abstract

Disease-causing parasites and pathogens play a pivotal role in intergroup behavior. Previous studies have suggested that the selection pressure posed by pathogen threat has resulted in in-group assortative sociality, including xenophobia and in-group favoritism. While the current literature has collated numerous studies on the former, strikingly, there has not been much research on the relationship between pathogen threat and in-group cooperation. Drawing upon prior studies on the function of the behavioral immune system (BIS), we argued that the BIS might facilitate cooperation with in-group members as a reactive behavioral immune response to pathogen threat. More specifically, we held that individuals might utilize cooperative behavior to ensure that they can receive social support when they have contracted an infectious disease. We reviewed existing findings pertaining to the potential role of the BIS in in-group cooperation and discussed directions for future studies.

Human life is rife with diverse natural threats such as disease-causing parasites and the paucity of resources. Evolutionary biologists have posited that such harsh environments favor cooperation (Jetz and Rubenstein, 2011; Smaldino et al., 2013; Lukas and Clutton-Brock, 2017; Liu et al., 2020), and researchers from various disciplines have demonstrated that the selection pressure posed by nature has contributed to the evolution of human cooperation. Recently, humans have faced the global pandemic (COVID-19), and this has drawn scholarly attention toward one of the most influential natural threats, pathogen threat. Intriguingly, however, while it would be of great importance to elucidate how pathogen threat would shape cooperative behavior, there has not been much research on it. Thus, we shall review existing evidence pertaining to the relationship between pathogen threat and group cooperation, and discuss future directions for this burgeoning field of research.

## The Behavioral Immune System And Assortative Sociality

Pathogens have acted as strong selection pressure throughout human evolution (Dobson and Carper, 1996; Wolfe et al., 2007), and humans have developed the biological immune system, which defends themselves against such external threats. Lately, researchers have argued that people have also evolved to have psychological mechanisms that facilitate behavioral defense against pathogens, the behavioral immune system (the BIS: Schaller, 2006, 2011; Schaller and Park, 2011). The BIS detects cues of infectious diseases and encourages behavioral responses to protect themselves from pathogens. Given that the risk of overlooking pathogen cues looms much larger than that of mistakenly responding to non-disease cues (Haselton and Buss, 2000), the BIS has become hypersensitive to external stimuli and reacts to diverse disease-irrelevant cues such as the elderly (Duncan and Schaller, 2009; Miller and Maner, 2012), obesity (Park et al., 2007; Miller and Maner, 2012), facial birthmarks (Ackerman et al., 2009), and disabilities (Park et al., 2003; Lund and Boggero, 2014; Dawydiak et al., 2020). Importantly, the BIS holds the functional flexibility such that the sensitivity to and the intensity of behavioral responses toward potential disease cues depend on the perceived vulnerability to infections (Mortensen et al., 2010; Miller and Maner, 2011; Tybur and Lieberman, 2016).

Another important feature of the BIS is assortative sociality. Based on the basic tenets of the BIS, Fincher and Thornhill (2012) have posited that disease-causing parasite stress has shaped ancestrally adaptive intergroup behavior such as avoidance of out-group members and social cohesion with in-group members. In the present article, we refer to them as out-group- and in-group-oriented minds, respectively. Since the relationship between the out-group-oriented mind and the BIS has been extensively reviewed elsewhere (Kusche and Barker, 2019), we shall briefly overview existing research on it and focus more on how the BIS influences the in-group-oriented mind.

## The Bis And Out-Group-Oriented Mind

In the context of the BIS, out-group-oriented mind manifests itself as behavioral avoidance and negative attitudes (i.e., xenophobia) toward out-group members; Fincher and Thornhill (2012) argued that people would avoid others outside of their natal area (i.e., out-group members) because they would be likely to carry unfamiliar pathogens (Fincher and Thornhill, 2008, 2012; cf., De Barra and Curtis, 2012; Karinen et al., 2019; Bressan, 2021) and/or violate local norms that have been partly established to deal with pathogen threats (Murray et al., 2011; cf., Aarøe et al., 2017; Karinen et al., 2019). Since negative attitudes toward out-group members underlie various social issues such as opposition to immigration, numerous studies have been conducted to investigate how the BIS would shape attitudes toward out-group members, especially immigrants (Faulkner et al., 2004; Navarrete and Fessler, 2006; Huang et al., 2011; Klavina et al., 2011; Krings et al., 2012; Hodson et al., 2013; Kim et al., 2016; Aarøe et al., 2017; Oaten et al., 2017; Laakasuo et al., 2018; Ji et al., 2019; Karinen et al., 2019; Zakrzewska et al., 2019; Meleady et al., 2021). Previous studies, for instance, have documented the association between pathogen concern and negative attitudes toward immigrants (Faulkner et al., 2004; Aarøe et al., 2017; Laakasuo et al., 2018; Ji et al., 2019). Moreover, experimentally induced pathogen threat has also been found to increase negativity against immigrants (Faulkner et al., 2004; Huang et al., 2011). Overall, the out-group-oriented-mind and resulting behavioral and affective responses function as a proactive defense system against pathogens, although there are different theories accounting for why out-groupness has become a trigger of behavioral immune responses.

## The Bis And In-Group-Oriented Mind

Fincher and Thornhill (2012) also held that pathogen prevalence led people to build a supportive social network for coping with infectious disease in the in-group. Supporting this, previous studies have found that national and regional pathogen prevalence is positively associated with various forms of in-group-oriented mind such as collectivism (Fincher et al., 2008; Thornhill et al., 2010; Cashdan, 2012; also see Cashdan and Steele, 2013). Wu and Chang (2012) also revealed the association between perceived vulnerability to disease and conformity. Moreover, Van Leeuwen et al. (2012) showed that individuals in historically pathogen-rich areas tended to endorse group-binding moral values. This finding was further buttressed by Makhanova et al. (2019) who found that trait pathogen sensitivity was positively associated with group-binding moral values (i.e., sanctity, loyalty, and authority). Overall, these correlational findings support the notion that pathogen threats have fostered several forms of the in-group-oriented mind.

As suggested by Fincher and Thornhill (2008), the endorsement of in-group-oriented values would help individuals solicit social support from in-group members to deal with disease infections. Yet, it should be noted that individuals do not need such support to avoid diseases. Moreover, as in-group members can be a primary source of infection to which the BIS responds with avoidance (Wu et al., 2015, 2019; van Leeuwen and Petersen, 2018), active contact with them can be counterproductive as a means for infection prevention. Thus, these together suggest that an in-group-oriented mind would act as a reactive defense system which operates in response to infection, rather than a proactive defense system (i.e., infection prevention). Accordingly, in-group-oriented behavioral immune responses would be triggered in situations where individuals have contracted infectious diseases, or they suspect they have. Thus, in-group- and out-group-oriented minds, characterized as reactive and proactive defense systems, respectively, should be distinct adaptation strategies and discussed separately.

Given that the BIS would facilitate in-group-oriented mind as a reactive response to pathogen threat, it should be carefully considered whether commonly used disease threat priming methods would be appropriate to study the relationship between pathogen threat and in-group-oriented behavior. Previous studies typically employed slide shows of disease-relevant sentences (e.g., Navarrete and Fessler, 2006) and a bogus article highlighting the threat of an epidemic (e.g., Miller and Maner, 2011). These manipulations cannot distinguish between two types of pathogen concerns: concern that one might contract or might have already contracted an infectious disease. Does the existing pathogen threat manipulation sufficiently trigger in-group-oriented behavioral responses? The current literature suffers from mixed results as to whether such experimental manipulations lead to increased in-group-oriented minds, pointing to the potential importance of separating different types of pathogen concerns.

Navarrete and Fessler (2006), for instance, used disgust-eliciting sentences to induce pathogen threat and demonstrated that individuals who were exposed to the priming stimuli exhibited more positive attitudes toward an in-group member than those who were not. In line with this, Wu and Chang (2012) employed photos depicting disgust-eliciting images (e.g., maggots and gory wounds) and showed that individuals who saw the photos reported more conforming tendencies than those who did not. Thus, there is some evidence suggesting that such priming methods would elicit in-group-oriented behavioral immune responses.

Contrastingly, Makhanova et al. (2019) revealed that while trait pathogen sensitivity was associated with group-binding moral values, experimentally induced pathogen threat did not influence the endorsement of such values. They conducted four studies with different experimental manipulation of pathogen threat (e.g., showing photos of visibly ill people and having participants read an article about the threat of a flu epidemic), and their meta-analysis on these studies did not found evidence that situationally induced pathogen threat increased the endorsement of in-group binding value systems. This finding apparently speaks to the crucial feature of in-group-oriented behavior as a reactive behavioral immune response, suggesting that the experimental manipulations, which arguably prime the feeling of being threatened by a possibility that they would catch a disease, would not trigger in-group-oriented behavior. Thus, the existing disease manipulation might not be appropriate to study the reactive behavioral immune responses.

Overall, previous studies have collated mixed results regarding the causal relationship between pathogen threat and in-group-oriented mind, whereas correlational studies have yielded consistent results. Presumably, the mixed findings are partly due to the nature of the manipulations, which do not specifically prime pathogen concern that one might have contracted a disease and need social support. Thus, it would be of vital importance for future studies to carefully consider a choice of manipulation stimuli and ensure that they establish an experimental setting that should trigger in-group-oriented behavioral responses.

## The Bis And In-Group Cooperation

We have so far discussed the endorsement of in-group-oriented values associated with the BIS as a reactive defense mechanism, and have argued that they function as an adaptive strategy to solicit social support from in-group members. However, it remained uncertain whether the endorsement of such in-group binding values would, in fact, earn help from others. Addressing this, social and evolutionary psychologists have revealed that cooperation indeed results in receiving social support from other in-group members. Thus, in-group cooperation can be a behavioral immune response to pathogen threat.

Yamagishi and colleagues conducted a series of experiments and posited that in-group cooperation had evolved to receive social support from other in-group members (Yamagishi et al., 1999; Yamagishi and Kiyonari, 2000). Importantly, they also found that individuals displayed in-group favoring tendencies only when they could expect that in-group cooperation would earn social support (i.e., reciprocity) from other in-group members (Yamagishi et al., 1999; Yamagishi and Kiyonari, 2000; Yamagishi and Mifune, 2008; Mifune et al., 2010). Thus, these findings suggest that it is plausible that the in-group-oriented mind and resulting behaviors have been acquired as an adaptation to mutual cooperation within the in-group. Considering this, it would be likely that the BIS promotes cooperation with in-group members as a means to secure social support.

However, there have been several studies showing that the BIS would rather reduce in-group cooperation under pathogen threat; Wu and colleagues have claimed that when in-group members are perceived to be primary sources of infection, the BIS encourages individuals to avoid and develop negative in-group attitudes (Wu et al., 2015, 2019). In addition, van Leeuwen and Petersen (2018) provided further evidence that the BIS would facilitate in-group avoidance; they had American and Indian participants and presented them with faces of their national in-group and out-group members with or without an explicit pathogen cue (a sore on the cheek). Participants were asked to indicate to what extent they would be comfortable with shaking hands and sitting next to these targets. Their study revealed that both Americans and Indians felt less comfortable with contact with others with the pathogen cue than those without it, regardless of their group membership. Therefore, the proactive behavioral immune response (i.e., behavioral avoidance) triggered by pathogen threat would apply to in-group members, and this would hinder in-group cooperation.

Yet, we would like to note that van Leeuwen and Petersen's (2018) finding should be treated with caution; Bressan (2021) reanalyzed van Leeuwen and Petersen's (2018) data and revealed that individuals were less comfortable with contact with dissimilar others than similar others and the presence of an explicit pathogen cue exacerbated the contrast. Thus, the data was not in fact in favor of the notion that the proactive immune system would encourage individuals to avoid in-group members with a disease cue.

When infectious diseases are rampant within the group (i.e., in an epidemic or a historically pathogen-rich area), it would be more likely that individuals contract them from other in-group members. Accordingly, it can be assumed that individuals would avoid in-group members, even when they do not possess any explicit pathogen cues. Consistently with this, Aarøe et al. (2016) reported a negative correlation between pathogen disgust sensitivity and trust, and the strength of the relationship did not substantially differ depending on whether participants were asked about others in general or in-group members (i.e., neighbors). This points to the possibility that pathogen threat leads the BIS to signal behavioral avoidance toward in-group members without explicit pathogen cues.

Related to this, Tybur et al. (2020) revealed behavioral immune tradeoffs; they found that the extent to which individuals felt comfortable with potentially infectious contact with others was moderated by the value they place on others. More specifically, individuals were more comfortable with contact with highly valued others, compared to those who were less valued, implicating that the reactive behavioral immune response could be compromised by interpersonal value. They employed a welfare-trade-off ratio (WTR) as an indicator of interpersonal value, and this predicted contact comfort.

WTR represents the willingness to tradeoff personal benefits to enhance the welfare of others, and previous studies revealed its pivotal role in cooperative behavior (Hartig, 2011; Smith et al., 2017). WTR is relatively high for in-group members compared to out-group members (Hall et al., 2021), and, therefore, individuals would not avoid in-group members. Accordingly, the proactive behavioral immune response might not be a barrier to in-group cooperation. Overall, there remains a debate as to whether the proactive defense system would target in-group members, and future studies should investigate whether and when the BIS would facilitate behavioral avoidance toward in-group members and reduce in-group cooperation.

Regarding the realm of others whom individuals are compelled to avoid under pathogen threat, there is an alternative scenario to consider, where existing group boundaries and memberships would no longer guide cooperation; Makhanova et al. (2015) revealed that trait pathogen concern and situational pathogen threat interactively influenced social categorization itself. More specifically, they found that white Americans with a low propensity to avoid germs tended toward categorizing elderly people who hold a pathogen cue (i.e., age) as out-group members, regardless of their racial group membership, when they were primed with pathogen threat. However, they did not display such a tendency when the disease priming was not administered. On the other hand, those with high trait germ aversion were more likely to categorize elderly black individuals as out-group members, whether or not they received disease-eliciting priming stimuli. In other words, situational and trait disease concerns interact to (re-)define the most meaningful group boundaries. Thus, under pathogen threat, existing group categorizations might not act as a reference for the BIS, and the evaluation of whether or not individuals should avoid others would be calibrated person-to-person basis, for instance, utilizing perceived physical similarity with others (Bressan, 2021). In this case, disease threat might produce small clusters of new, cohesive groups in which individuals would not avoid each other but provide extensive social support. Future studies should, therefore, carefully examine whether it is a mere group membership, *per se*, which is associated with behavioral immune responses, and we believe the minimal group paradigm (Tajfel et al., 1971) would be helpful in doing so.

## Conclusion And Future Directions

We first pointed to the crucial yet overlooked difference between in-group favoritism and xenophobia driven by the BIS—based on the theorizing by Fincher and Thornhill (2012), we argued that in-group favoritism is a reactive, rather than proactive defense against pathogen threat and discussed in what circumstances pathogen threat would promote in-group cooperation. We would like to emphasize again that despite the practical and theoretical importance of the elucidation of the implications of the BIS for cooperation, no studies have directly addressed this so far. Thus, future studies should first investigate how trait and experimentally induced pathogen concerns would influence cooperative behavior, for instance, using economic game paradigms (Thielmann et al., 2021). For the experimental manipulation of disease salience, existing priming methods might not be suitable to elicit in-group-oriented behavioral immune responses, and a new experimental paradigm might be necessary.

Overall, the current empirical literature calls for studies on the BIS and in-group cooperation, leaving us several promising directions to further elucidate the role of pathogen threat in in-group cooperation. In-group cooperation has been typically discussed in relation to another evolutionary mechanism, the reputation system (Yamagishi et al., 1999; Yamagishi and Mifune, 2008; Mifune et al., 2010), and the elucidation of the role of the BIS would allow us to examine whether and how the two distinct evolutionary mechanisms together shape cooperation, assuming that the BIS has contributed to the evolution of in-group cooperation. Thus, further research will certainly help researchers address broader and more general issues such as the evolution of group cooperation.

On a final note, we would like to discuss two alternative scenarios, which future empirical work may reveal. First, despite that we have described in-group cooperation as a reactive behavioral immune response, in-group cooperation might function as part of the proactive behavioral immune system. Especially in pathogen-rich regions, it would be likely that pathogen treats are always, to some extent, salient and this would encourage individuals to display cooperation to ensure they receive support from other in-group members when necessary. Thus, although cooperation often requires physical contact and may not be an effective strategy as a proactive defense against diseases, there is no denying that in-group cooperation might be enforced by the proactive behavioral immune system. Second, there remains the possibility that the BIS might not facilitate in-group cooperation; researchers have argued that expression of disease symptoms (i.e., sickness) would signal the need for social support, and it might be sufficient to solicit help from in-group members, at least, among humans (Tiokhin, 2016). Thus, it would not be surprising if future studies discovered evidence for the absence of the association between the BIS and in-group cooperation. Then, presumably, the reactive defense against diseases would be treated by different evolutionarily acquired systems such as the reputation system.

## Author Contributions

HI and NM conceived of the presented ideas. HI wrote the first draft of the manuscript. All authors equally contributed to finalizing the manuscript.

## Conflict of Interest

The authors declare that the research was conducted in the absence of any commercial or financial relationships that could be construed as a potential conflict of interest.
